# Determinants of the CmoB carboxymethyl transferase utilized for selective tRNA wobble modification

**DOI:** 10.1093/nar/gkv206

**Published:** 2015-04-08

**Authors:** Jungwook Kim, Hui Xiao, Junseock Koh, Yikai Wang, Jeffrey B. Bonanno, Keisha Thomas, Patricia C. Babbitt, Shoshana Brown, Young-Sam Lee, Steven C. Almo

**Affiliations:** 1 Department of Biochemistry, Albert Einstein College of Medicine, Bronx, NY 10461, USA; 2 Department of Pathology, Albert Einstein College of Medicine, Bronx, NY 10461, USA; 3 Laboratory of Cell Biology and Howard Hughes Medical Institute, The Rockefeller University, New York, NY 10065, USA; 4 Chemical Biology Program, Broad Institute of Harvard and MIT, Cambridge, MA 02142, USA; 5 Department of Bioengineering and Therapeutic Sciences, University of California at San Francisco, San Francisco, CA 94158, USA; 6 Department of Biology, Johns Hopkins University, Baltimore, MD 21218, USA; 7 Department of Physiology & Biophysics, Albert Einstein College of Medicine, Bronx, NY 10461, USA

## Abstract

Enzyme-mediated modifications at the wobble position of tRNAs are essential for the translation of the genetic code. We report the genetic, biochemical and structural characterization of CmoB, the enzyme that recognizes the unique metabolite carboxy-*S*-adenosine-L-methionine (Cx-SAM) and catalyzes a carboxymethyl transfer reaction resulting in formation of 5-oxyacetyluridine at the wobble position of tRNAs. CmoB is distinctive in that it is the only known member of the SAM-dependent methyltransferase (SDMT) superfamily that utilizes a naturally occurring SAM analog as the alkyl donor to fulfill a biologically meaningful function. Biochemical and genetic studies define the *in vitro* and *in vivo* selectivity for Cx-SAM as alkyl donor over the vastly more abundant SAM. Complementary high-resolution structures of the apo- and Cx-SAM bound CmoB reveal the determinants responsible for this remarkable discrimination. Together, these studies provide mechanistic insight into the enzymatic and non-enzymatic feature of this alkyl transfer reaction which affords the broadened specificity required for tRNAs to recognize multiple synonymous codons.

## INTRODUCTION

All organisms contain fewer than 61 unique tRNA species, precluding a direct one-to-one mapping between anticodons and sense codons. This incongruence requires some anticodons to recognize multiple codons for complete translation of the genetic code. The needed degeneracy derives from non-canonical Watson–Crick pairings between the 3′ nucleotide of a codon triplet and the ‘wobble’ nucleotide at the 5′-end of an anticodon (1). These wobble nucleotides are the targets for numerous enzyme-mediated modifications, which are important for fundamental aspects of translation, including the broadened specificity required for degenerate codon recognition (2–4), ribosome binding (5,6), reading frame maintenance (7) and translocation (8). Wobble uridines are almost always modified and 5-oxyacetyluridine (cmo5U) is observed in isoacceptors of Ala-, Ser-, Val-, Thr- and Pro-specific tRNAs in *Escherichia coli* (9), and is a widespread feature in the Gram-negative proteobacteria. It was shown that cmo5U-containing tRNAs specific for Ala, Val and Pro can each recognize four degenerate codons in *Salmonella enterica* (10). Direct crystallographic analysis revealed the structural and chemical features responsible for the expanded codon recognition properties exhibited by cmo5U (11), including an intramolecular hydrogen between the ether oxygen of the oxyacetyl moiety and the 2′-hydroxyl of U33, which promotes an anticodon stemloop conformation that supports degenerate binding to multiple codons.

**Figure 1.**
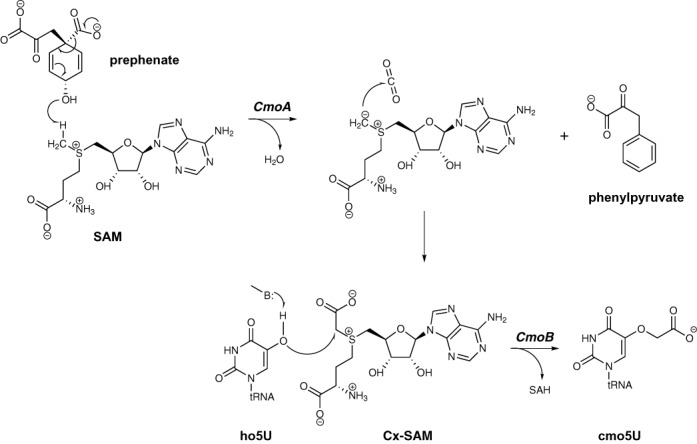
Proposed chemical mechanism for cmo5U biosynthesis. The 5-hydroxyl group on ho5U becomes deprotonated by a general base (denoted as B). The activated nucleophile then attacks the *S*-carboxymethyl of Cx-SAM, derived from the CmoA-mediated reaction with SAM and prephenate.

While the determinants of cmo5U function are well defined, clarity has only recently begun to emerge regarding the relevant biosynthetic transformations leading to the cmo5U modification. Chorismate, or a related metabolite, had been implicated in the biosynthesis of cmo5U in *E. coli*, although the precise role of this compound in cmo5U formation was not clear (12). Subsequent mutagenesis experiments in *S. enterica* identified two members of the SDMT superfamily, CmoA and CmoB, as important components of the cmo5U biosynthetic pathway. *cmoA*-deficient mutants accumulate 5-hydroxyuridine (ho5U) and 5-methoxyuridine (mo5U), while *cmoB* mutants exclusively accumulate ho5U (13). The observation of mo5U was taken as evidence of its role as an obligate intermediate preceding cmo5U formation, which confounded the mechanistic interpretation of the cmo5U biosynthetic pathway. Recently, we demonstrated that cmo5U modification requires the previously unknown metabolite carboxy-*S*-adenosine-L-methionine (Cx-SAM), which is generated by the CmoA-catalyzed conversion of prephenate and SAM to yield Cx-SAM and phenylpyruvate. This remarkable reaction proceeds via a unique SAM-based sulfur-ylide intermediate, which captures the carbon dioxide moiety liberated as the consequence of decarboxylation of prephenate (14). Cx-SAM is subsequently utilized in the CmoB-catalyzed carboxymethylation of ho5U-containing tRNAs to yield the mature cmo5U-modified tRNAs (14), with no involvement of a mo5U intermediate (Figure 1). This transformation is notable as Cx-SAM must compete with SAM, which is one of the most abundant cofactors in nature (i.e. 180 μM in *E. coli*) (15–17). Hydroxylation of the wobble uridine is catalyzed by an as-yet-unidentified enzyme.

To further define the biosynthetic pathway and mechanistic details associated with cmo5U tRNA modification, we report the X-ray crystal structures of apo- and Cx-SAM bound CmoB, which represents the first instance of an enzyme that utilizes a naturally occurring SAM-derivative in a biologically meaningful alkyl transfer reaction. The structures of CmoB provide valuable insight into the physical and chemical determinants required for the discrimination of Cx-SAM over SAM, which is needed to achieve the high selectivity observed in tRNA modification. We have also confirmed the *in vivo* and *in vitro* activities of CmoB in the Cx-SAM-dependent carboxymethylation of tRNA, supporting the functional assignment of CmoB as a novel carboxymethyl transferase. Finally, we have demonstrated that CmoB is responsible for the mechanistically confounding SAM-dependent formation of mo5U observed in the absence of Cx-SAM.

## MATERIALS AND METHODS

For crystallization and structural determination, please refer to Supplementary methods.

### Cloning and protein purification

The *cmoB* gene was amplified from genomic DNA of *E. coli* BL21 by polymerase chain reaction (PCR), cloned into LIC-pET30a (Novagen) and verified by DNA sequence analysis (Genewiz). *Escherichia coli* BL21 (DE3) cells (Invitrogen) were transformed with vectors harboring the *cmoB* gene, grown in lysogeny broth (LB) containing 50-mg/ml kanamycin at 37°C and induced with 0.5-mM Isopropyl β-D-1-thiogalactopyranoside (IPTG) at an OD_600_ of ∼1. Cells were incubated overnight at 25°C and harvested by centrifugation. Cell pellets were resuspended and lysed with Bugbuster (Novagen) at room temperature for 30 min, the lysates centrifuged at 41 400*g* for 30 min and the supernatants applied to Ni-agarose (Qiagene) columns pre-equilibrated with buffer A containing 20-mM Tris-HCl, pH 8.5 and 150-mM KCl. The recombinant protein was eluted with 150-mM imidazole in buffer A and the affinity tag removed by thrombin (Novagen) cleavage at 4°C overnight. The protein was further purified by anion exchange chromatography on a MonoQ column (GE) equilibrated with buffer A and eluted with a linear gradient of buffer B (20-mM Tris-HCl, pH 8.5 and 1-M NaCl). Final purity was over 95% as verified by sodium dodecyl sulphate-polyacrylamide gel electrophoresis (SDS-PAGE) analysis. The yield was quantitated using an extinction coefficient of ϵ_280_ = 72.5 cm^−1^ mM^−1^, as calculated from the amino acid sequence.

For complementation assays, the wild-type *cmoB* gene was cloned into plasmid pQE30a (Qiagen) between the BamHI and HindIII sites. The *cmoB* mutants were generated by QuikChange (Stratagene) using the plasmid of the wild type as the template for PCR. The recombinant mutant CmoB proteins were prepared by subcloning the mutant *cmoB* gene into LIC-pET30a vector and the purification of the mutants was identical to that of the wild type described above.

### Purification of selenomethionine-substituted CmoB

*Escherichia coli* strain B834 (Novagen) was transformed with the LIC-pET30a-CmoB expression vector, grown in selenomethionine-containing media (Molecular Dimensions) at 37°C, induced with 0.5-mM IPTG when the OD_600_ reached ∼1 and further incubated overnight at 25°C. The selenomethionyl-substituted protein was purified in a fashion identical to that described for the native protein.

### Chemical synthesis of Cx-SAM

*S*-Adenosyl-L-homocysteine (15 mg) was dissolved in 2.5 ml of 150-mM ammonium bicarbonate. To this solution, 2-iodoacetatic acid (500 mg) was added. The mixture was incubated at 37°C for 18 h. After the reaction was completed, 60-ml methanol was added and the mixture incubated at 4°C overnight. Precipitates were collected by centrifugation at 4°C (2000*g* for 30 min), washed twice with ice-cold methanol to yield 2.7-mg product Cx-SAM. The product was dissolved in the appropriate solvent for further experiments. The concentration of Cx-SAM was determined spectroscopically, assuming an extinction coefficient of SAM (ϵ_260_ = 15.4 cm^−1^ mM^−1^).

### cmoB complementation and liquid chromatography-tandem mass spectrometry analysis

*cmoB*-deficient *E. coli* cells (from the KEIO collection (18)) were transformed with plasmids bearing wild-type or mutant *cmoB*, and selected with ampicilin. Transformed cells were grown in 50-ml LB media at 37°C and induced at an OD_600_ of ∼1.0 with 0.5-mM IPTG. Cells were incubated at 25°C and harvested by centrifugation. An aliquot of 10 ml of cells was withdrawn at 3-, 6- and 24-h time points after induction of CmoB expression. Total RNA was extracted, digested and analyzed as described previously with slight modification (14). In brief, cellular RNA was purified with Trizol (Ambion) according to the manufacturer's instruction, followed by incubation with 0.1-mg/ml P1 nuclease at 60°C for at least 1 h to convert polynucleotides into 5′-nucleotide monophosphates. The hydrolyzed nucleotide samples were analyzed using liquid chromatography-tandem mass spectrometry (LC-MS) (12T Agilent IonSpec FT-ICR-MS) in negative ion mode. An aliquot of 50-μl sample was injected onto the HPLC column (Waters Symmetry C18 Column, 100 Å, 3.5 μm, 2.1 mm X 150 mm) coupled to a Shimadzu HPLC (Shimadzu, Kyoto Japan), with two LC-10AD pumps, to generate a gradient with a 50-μl·min^−1^ flow rate. Solvent A was 5% acetonitrile in H_2_O, 0.1% formic acid (FA); solvent B consisted of 95% acetonitrile in H_2_O, 0.1% FA. After desalting for 5 min with 2% B, the nucleotides were eluted at 50 μl·min^−1^ with a 2–10% gradient for 1 min, 10–20% for 30 min and 20–95% for 5 min.

### *In vitro* LC-MS-based assay

The assay solution for SAM-dependent mo5U formation contained 10-mM Tris-HCl (pH 8.0), 4-mM MgCl_2_, 0.2-mM SAM and 20–25 μg (∼20 μM) of total tRNA prepared from *cmoB*-mutant *E. coli* cells in 50 μl. Reaction conditions for Cx-SAM-dependent cmo5U formation assay were identical, with the exception that10-μM Cx-SAM was used in place of SAM. Assays were initiated by addition of 0.1–2-μM CmoB to the above mixtures and subsequently incubated at the room temperature. The reaction was terminated by the addition of 0.1-mg/ml P1 nuclease, followed by incubation at 60°C for at least 1 h. An assay sample quenched immediately after initiation resulted in no detectable formation of cmo5UMP, validating the effectiveness of the quenching method. Hydrolyzed nucleotides were directly applied to HPLC without further processing for LC-MS analysis as described above.

### LC-MS/MS analysis of nucleotides

Approximately 20 μg of the P1 nuclease-digested RNAs from wild-type or mutant *E. coli* (*ΔcmoA*, Δ*aroC* or Δ*cmoB*) were injected onto the aforementioned HPLC system coupled to an LTQ - Orbitrap Velos mass spectrometer (Thermo Fisher Scientific, Waltham, CA, USA) for MS/MS analysis in the negative mode to identify the modified nucleotides. Buffers and gradient were identical to those described above. To identify mo5UMP and cmo5UMP, the masses of these two nucleotides (calculated m/z = 353.04 and 397.03, respectively) were included in the parent ion inclusion list for MS/MS. These two masses were monitored in each scan on the Orbitrap MS with a resolution (*m/Δm*) of 60 000. For structure confirmation, collision induced dissociation MS/MS spectra were acquired only when these two nucleotides were detected with the correct accurate mass. MS/MS was performed using an isolation width of 2 Da; normalized energy of 35%; activation time of 30 ms and a minimum signal intensity of 200 counts. Dynamic exclusion was enabled with an exclusion duration of 90 s.

### Time course of SAM-dependent mo5U modification

The assay was initiated by mixing 10-mM Tris (pH 8.0), 4-mM MgCl_2_, 140-μM [^14^CH_3_]-SAM (Perkin Elmer), 100 μg of total tRNA prepared from *cmoB*-deficient cells and 2-μM CmoB in 50 μl. In competition assays to measure the effect of Cx-SAM on methylation, 10-μM Cx-SAM was added to the above mixture. An aliquot of 15 μl was removed at 15, 30 and 45 min after initiation of the assay and quenched with 0.5-ml 0.5-M sodium acetate (pH 4.0). RNA was washed with 0.2-M ammonium acetate (pH 6.0) using an MWCO 10-kDa filter by centrifugation. This washing step was repeated two more times to remove unreacted ^14^C-SAM. RNA was then spotted on a DE81 filter (GE) and air-dried for 20 min. Images of the radioactive tRNA were recorded on a phosphorimaging plate (Molecular Dynamics) for 2 days and analyzed using a Molecular Dynamics Storm 860 PhosphorImager System with ImageQuant software.

### Measurement of cellular concentration of Cx-SAM

The cellular concentration of Cx-SAM was quantified with an isotope ratio-based approach (17), using ^15^N-labeled isotopic standard of Cx-SAM. The isotopic standard (chemical formula = C_16_H_22_^15^N_6_O_7_S, [M+H] = 449.1171) was prepared by methanol extraction of the ligand from the recombinant CmoA, which had been purified from cells grown in M9 media supplemented with ^15^NH_4_Cl as the sole nitrogen source, followed by HPLC purification as described previously (14). Approximately 6 × 10^10^ wild-type *E. coli K-12* cells grown in 10-ml LB media were pelleted by centrifugation. Metabolites were extracted with 1 ml of methanol:acetonitrile:water (40:40:20) with 0.1-mM FA, and 90 nM of an isotopic standard of Cx-SAM. After 20-min incubation on ice, the lysed cells were centrifuged at 16 000*g* for 20 min at 4°C. The sample was subjected to LC-MS analysis (Agilent 1200 HPLC coupled to LTQ Orbitrap Velos mass spectrometer; ESI positive ion mode detection; Phenomenex Luna NH2 column, bead size of 5mm, pore size of 100 Å, 150 × 2 mm) using a gradient from 75 to 2% of acetonitrile and 0.1% FA over 30 min. The amount of cellular Cx-SAM was quantified by comparing the intensities of chromatographic peaks with those from the isotopic standard, assuming the intracellular volume of *E. coli* is ∼1 μm^3^ (19,20).

### Isothermal titration calorimetry assay

CmoB was extensively dialyzed for 24 h against binding buffer (20-mM HEPES, pH 7.5) using a dialysis cassette with 10-kDa MWCO (Pierce). Lyophilized Cx-SAM was dissolved in the binding buffer at a final concentration of 1.3 mM. SAM was purchased from Sigma and dissolved in binding buffer at a final concentration of 10 mM. Prior to use, all titration samples were centrifuged for 20 min at 4°C. Isothermal titration calorimetry (ITC) experiments were performed at 15°C using an auto-ITC_200_ (MicroCal, LLC, Northhampton, MA, USA) in which the reaction cell (202.8 μl) containing CmoB (50 μM and 370 μM for Cx-SAM and SAM, respectively) was titrated with 40 × 1-μl injections of 1.3-mM Cx-SAM or 6.7-mM SAM with an equilibration time of 180 s. Raw injection traces were integrated over time, normalized per mol of ligand injected and corrected for the heat of dilution obtained from the plateau of a titration curve. Titration data were analyzed using a single-site model in Origin (version X) and Igor Pro 5/6. Initially, an unexpectedly high stoichiometry for Cx-SAM (*n* = ∼2) was obtained from fitting the titration data to a single-site model, suggesting that approximately a half of the ligand used in the assay might be inactive. Based on the chemical synthesis, the Cx-SAM utilized in the binding assay is a racemic mixture, of which only the S-isomer is capable of acting as a substrate/ligand for CmoB. Therefore, the stoichiometry was fixed at 1:1 and the activity of the ligand was introduced as a fitting parameter for Cx-SAM.

## RESULTS

### Overall structure of CmoB

**Figure 2.**
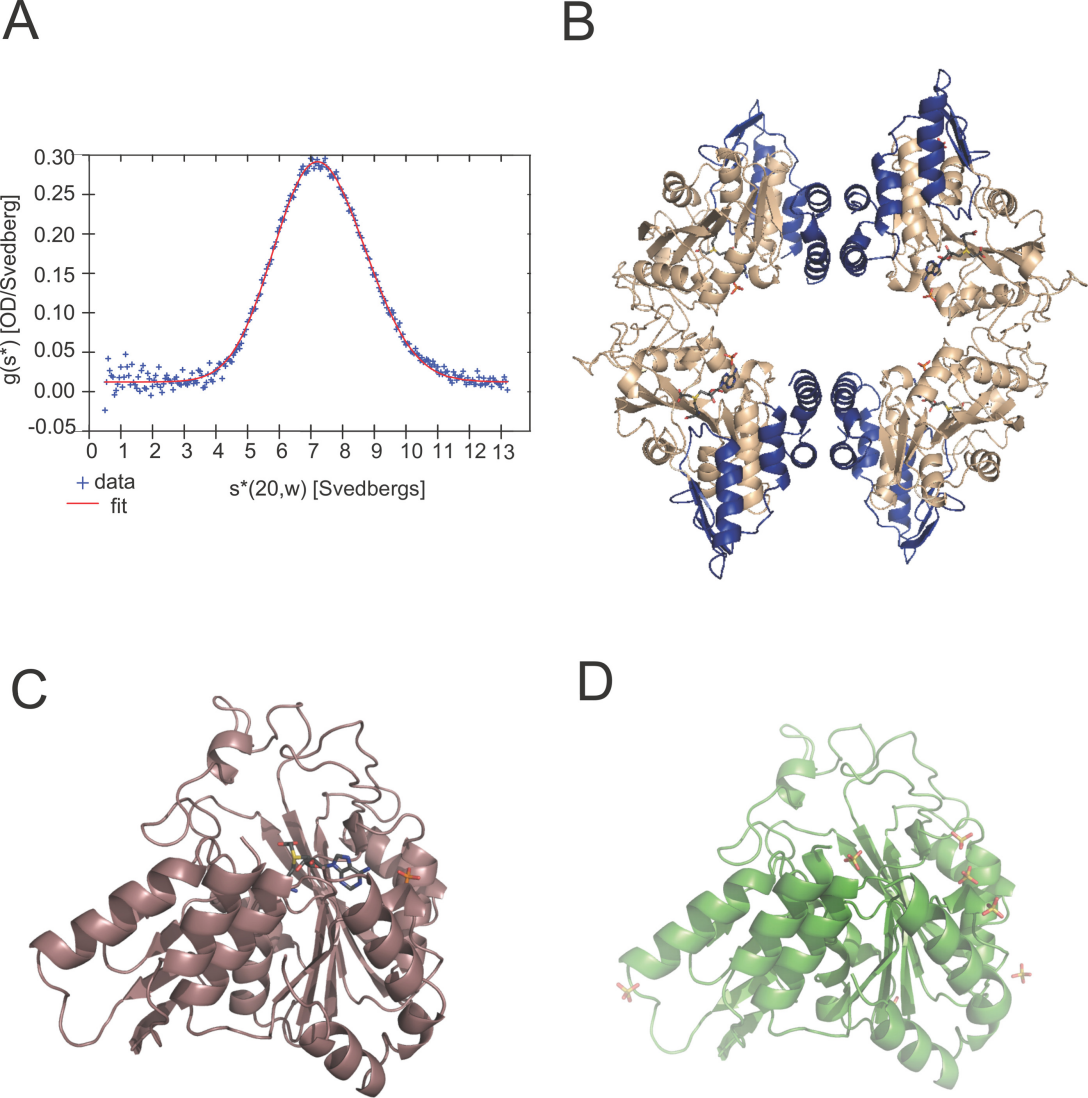
Overall structure of CmoB. (**A**) g(s*) distribution is fit with data from sedimentation velocity experiments for a single-species model. The best value for S_20,w_ is 7.27S, with an expected molecular weight of 154.28 kD. The calculated molecular weight of a monomer based on the amino acid sequences is 37.01 kD. (**B**) A tetramer of Cx-SAM bound CmoB, where the N-terminal 100 residues unique to CmoB are highlighted in blue. Cx-SAM and phosphate ions are displayed in sticks, where carbon atoms are in gray, oxygen atoms in red, nitrogen atoms in blue, sulfur atoms in yellow and phosphorus atoms in orange. (**C**) A monomer of Cx-SAM bound CmoB and (**D**) apo-CmoB.

Sedimentation velocity analysis of CmoB is consistent with a tetramer that is insensitive to Cx-SAM or SAM (Figure 2A). Experimental phases derived from selenomethionyl-substituted CmoB crystals were used to determine all three structures described herein (Supplementary Table S1). Cx-SAM bound CmoB structures were determined in two crystal forms, resulting in 10 independent copies of the complex (two tetramers in one crystal form, and one dimer in the other), while the apo structure contains a single dimer in the asymmetric unit (Figure 2). The dimers exhibit symmetry-related interactions that recapitulate the expected tetramer. Pairwise structural alignments of all 12 protomers (calculated on 319–323 Cα's) displayed RMSDs ranging from 0.10 to 0.51 Å, with the most variable region being a short loop composed of Gln-35, His-36 and Gly-37. This segment, which is more than 15 Å from the catalytic site, is disordered in seven out of the 12 chains in the three structures.

As predicted from sequence considerations, CmoB is composed of a Rossmann fold, coupled to a substrate-specific N-terminal domain of ∼100 residues that is unique to CmoB. A search for structural homologs with the DALI server (21) yielded hits containing a Rossmann fold, with the most similar being members of the SDMT superfamily: phosphoethanolamine N-methyltransferase (pdb code 4KRI; *z* = 18.0, RMSD = 3.3 Å, 26% identity over 190 residues), and a putative protein N-methyltransferase (pdb code 4INE; *z* = 17.9, RMSD = 3.2 Å, 21% identity over 186 residues). Superposition of these structures highlights the ∼100 N-terminal residues that are unique to CmoB (Supplementary Figure S1). A DALI search using residues 1–100 as the query only returns structures of CmoB orthologs. Residues 1–35 form a helix-turn-helix motif that contributes to the dimer interface and residues 36–100 possess considerable positive electrostatic potential suggesting a broad tRNA-binding platform. The Cx-SAM binding site is more exposed to solvent than observed for the Cx-SAM/SAM binding sites in CmoA and many other SAM-dependent methyltransferases, likely due to the need to accommodate bulky tRNA substrates.

### Atomic interactions between Cx-SAM and CmoB

**Figure 3.**
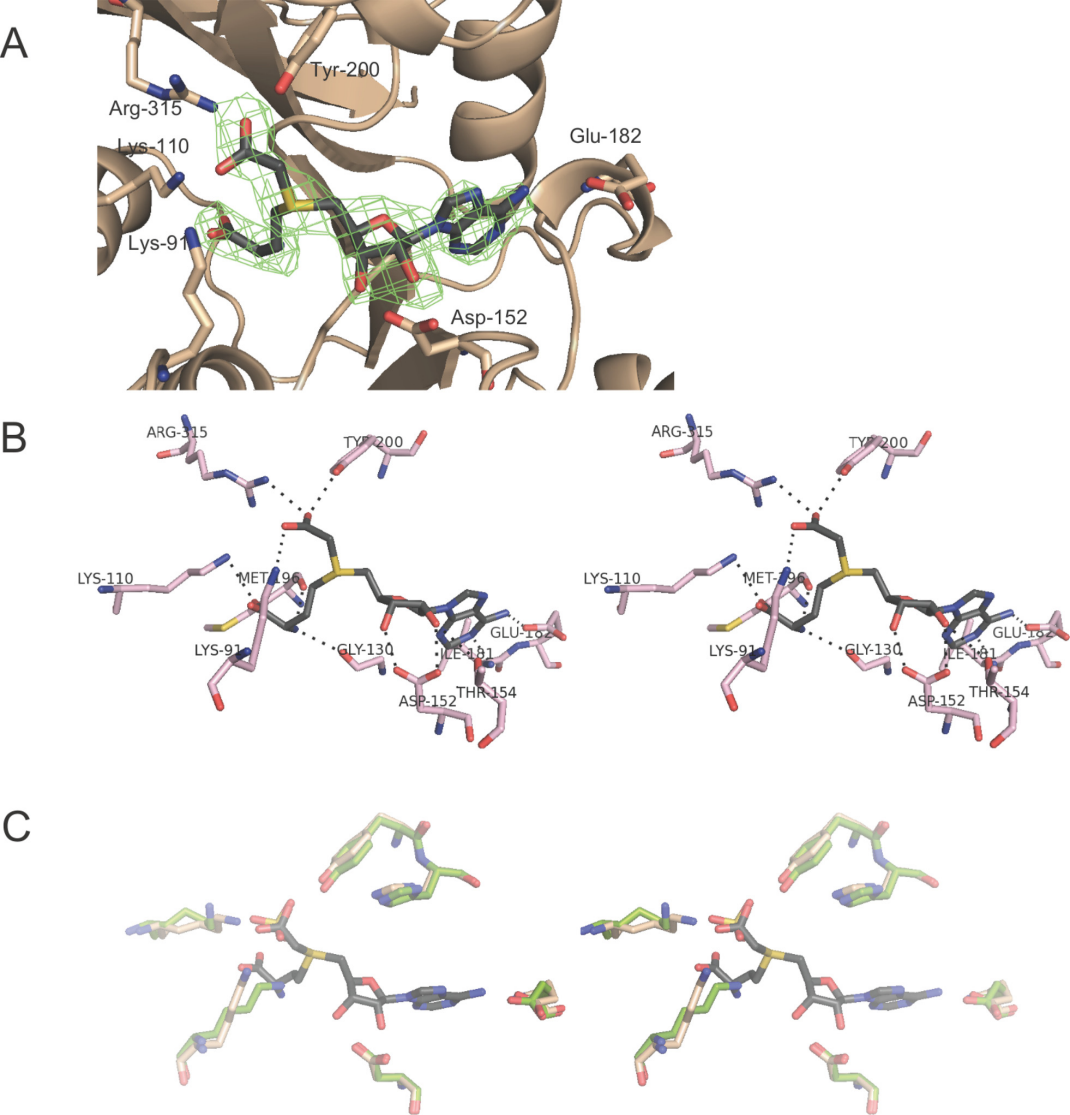
Cx-SAM binding site of CmoB. (**A**) *Fo-Fc* difference Fourier synthesis, calculated at 2.6-Å resolution without the ligand, contoured at 3σ around the modeled Cx-SAM ligand. (**B**) Polar interactions between Cx-SAM and CmoB. A cross-eye stereo presentation of Cx-SAM bound in the CmoB catalytic site, where amino acid residues within 3.5 Å of the ligand are shown. Hydrogen bonding and ionic interactions are depicted in dashed lines. (**C**) A cross-eye stereo presentation of an overlay of active sites of Cx-SAM bound and apo CmoB structures. Liganded CmoB residues are colored in light pink, and residues from the apo-structure are in green. An identical color scheme is applied to Cx-SAM and sulfate ion as in Figure 2.

Difference Fourier syntheses calculated after initial rounds of refinement, without the inclusion of ligand, exhibited features consistent with the binding of Cx-SAM in the catalytic site (Figure 3A). Continued refinement and modeling indicated that Cx-SAM is present at full occupancy and demonstrated similar atomic interactions in all 10 independent catalytic sites in both liganded structures.

Key polar interactions between CmoB and Cx-SAM in the crystalline state are summarized in Figure 3B. The side chains of Lys-91, Tyr-200 and Arg-315 interact with the carboxyl group of the *S*-carboxymethyl moiety of the Cx-SAM ligand. These three residues are nearly invariant among CmoB family members (815 out of 818 for Lys-91, 816 out of 818 for Tyr-200 and 814 out of 818 for Arg-315), and other amino acid residues composing the ligand binding site exhibit high conservation (Supplementary Figure S2). Three additional conserved residues within the CmoB family recognize the amino acid moiety of the ligand: the side chain of Lys-110 (815/818) forms polar interactions with the α-carboxy group, while the backbone carbonyl oxygen atoms of Gly-130 (817/818; the presence of a bulkier side chain would likely interfere with ligand binding) and Met-196 (606/818, where other instances are leucine) interact with the α-amino group of Cx-SAM. The side chain of Glu-182 (96% conservation) forms polar interactions with N6 of the adenine moiety and the side chain of Asp-152 (∼97% conserved in CmoB orthologs, and highly conserved in all members for SAM-dependent methyltransferase superfamily) interacts with the 2′- and 3′-hydroxyls. The side chain of Thr-154 (only 29.9% conservation) also forms a hydrogen bond with the 2′-hydroxyl of Cx-SAM.

The binding scheme employed by CmoB is quite different from that utilized by CmoA, where only Arg-199 contacts the carboxyl group of the *S*-carboxymethyl side chain of Cx-SAM via a bidentate interaction. In addition, the backbone carbonyl oxygen atom of Phe-133 forms a potential carbon-oxygen (CH···O) hydrogen bond (22) with the methylene hydrogen of the *S*-carboxymethyl group in the CmoA active site; however, no equivalent interaction is observed in CmoB. In contrast, backbone oxygen atoms of Met-196 and Gly-197 of CmoB form potential carbon–oxygen hydrogen bonding interactions with the other two methylene groups adjacent to the sulfonium center of Cx-SAM (Supplementary Figure S3A); these interactions are absent in the CmoA structure. Although Met-196 is somewhat variable, Gly-197 is highly conserved (816 out of 818), as the presence of a bulkier side chain would be predicted to interfere with Cx-SAM binding. Within the catalytic site of CmoB, Cx-SAM is highly exposed to the bulk solvent, whereas in CmoA it is substantially more buried (Supplementary Figure S3B). Considerable positive electrostatic potential surrounds the Cx-SAM binding pocket on the surface of the CmoB, consistent with the docking site of polyanionic tRNA molecules (Supplementary Figure S4A).

### Responsiveness to ligand binding

The overall fold of CmoB is essentially identical in all apo- and Cx-SAM bound structures (Figure 2). A total of 16 sulfate ions were modeled in the two independent copies of the apo-structure (seven at similar positions in both independent molecules and two additional sulfates unique to each of the molecules), which likely originated from the crystallization media. Of particular note are six sulfate ions, present in each independent molecule, located on the surface with positive electrostatic potential surrounding the catalytic site. In all subunits in both Cx-SAM bound structures, a feature modeled as a phosphate ion is present between two basic residues, Arg-202 and His-208 (98.3 and 67.1% conservation, respectively) (Supplementary Figure S4B), with one of the presumed sulfate ions from the crystallization media present at a similar position in the apo structure. This ion is the closest to the Cx-SAM ligand (∼5.6 Å from N6).

One of the sulfates modeled in the apo-structure occupies a site analogous to that of the carboxyl group of the*S*-carboxymethyl moiety in the Cx-SAM bound structures (Figure 3C). There are no major structural differences between the active sites of Cx-SAM bound and apo-structures, with the exception of Lys-91, which exhibits a shift of ∼4 Å. The side chain of Lys-91 contacts the *S*-carboxymethyl group in the Cx-SAM complex, while in the apo-structure it interacts with the backbone carbonyl of Gly130 and the sulfate that mimics the *S*-carboxymethyl moiety. Tyr-200 and Arg-315 also interact with this same sulfate ion in the apo-structure.

### *In vivo* activity of wild-type CmoB

**Figure 4.**
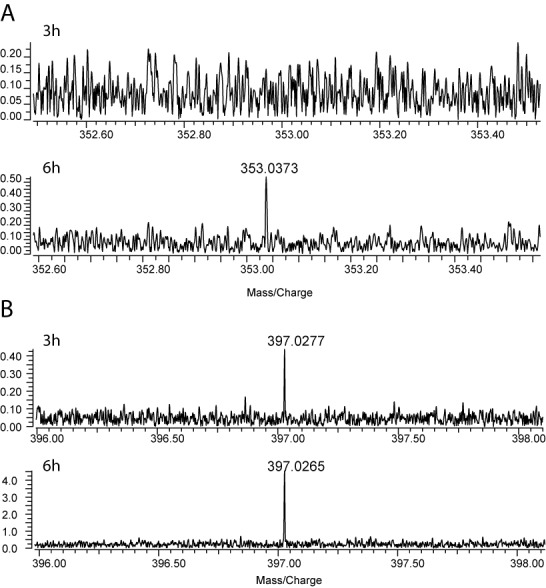
*In vivo* assay of cmo5U and mo5U formation by wild-type CmoB. Total RNA from Δ*cmoB E. coli* complemented with plasmid-encoded wild-type *cmoB* gene was extracted 3 and 6 h after induction with 0.5-mM IPTG, followed by P1 nuclease treatment and LC/MS analyses in negative ion mode. (**A**) mo5UMP was not detected after 3 h of induction (top), but was detected after 6 h (bottom), whereas (**B**) cmo5UMP was detected as early as 3 h after induction.

It was reported that total tRNA derived from *cmoA*-deficient mutants possessed both ho5U and mo5U, in contrast to wild-type total tRNA which possessed predominately cmo5U (13). The presence of small amounts of mo5U has also been reported previously from total RNA (23) and tRNA (24) of wild-type *E. coli*. When we examined P1 nuclease-treated total tRNA from wild-type *E. coli* by LC-MS/MS, a trace amount of mo5U was indeed detected in addition to the much more abundant cmo5U (Supplementary Figure S5). Enrichment of mo5U was observed in Δ*cmoA*- and Δ*aroC*-mutants, where *aroC* encodes the protein catalyzing the formation of chorismate, the immediate precursor of prephenate required for the CmoA-catalyzed transformation (Supplementary Figure S5). No significant amount of cmo5U was detected in these mutants, as these mutants are unable to synthesize Cx-SAM (12,13,25). Both mo5U and cmo5U were absent from Δ*cmoB*-mutant, in agreement with a previous report (13). These findings suggest that in addition to Cx-SAM-dependent cmo5U formation, CmoB catalyzes the SAM-dependent methylation of wobble ho5U in Gram-negative organisms, albeit with much lower efficiency. To investigate the *in vivo* generation of mo5U and cmo5U, modifications were monitored in *cmoB*-deficient cells complemented with a plasmid directing the inducible production of wild-type *cmoB*. Total RNA was extracted from cells at 3-, 6- and 24-h time points after the induction of CmoB expression, and nucleotides derived from P1 nuclease treatment were analyzed by LC-MS (Figure 4). Three hours post-induction of *cmoB* expression, cmo5U was detected in the sample, while no mo5U was observed. The MS peak corresponding to mo5U began to appear 6 h post-induction, confirming that CmoB is involved in the *in vivo* generation of both cmo5U and mo5U.

### *In vitro* activity of the wild-type CmoB

The *in vitro* activity of CmoB was examined with chemically synthesized Cx-SAM and ho5U-containing tRNAs prepared from *cmoB*-defective *E. coli* cells (Supplementary Figure S6). Incubation of 0.1-μM wild-type CmoB with 10-μM Cx-SAM resulted in detectable amounts of cmo5U after 9 min. Similar to the low *in vivo* activity, mo5U was only observed after 5 h when 0.2-mM SAM, a physiological concentration in *E. coli* (17), was used as alkyl donor instead of Cx-SAM, with 2-μM wild-type CmoB.

To determine whether SAM can efficiently compete with Cx-SAM *in vitro*, the time course of mo5U formation was monitored by incubating 140 μM [^14^CH_3_]-SAM, CmoB and ho5U-containing tRNAs, with or without unlabeled 10-μM Cx-SAM for 90 min. There was no detectable radioactive signal from tRNA when Cx-SAM was present in the assay mixture (Supplementary Figure S7), suggesting that rapid conversion of ho5U to cmo5U depleted available methyl acceptor sites before significant methylation occurred. To confirm that CmoB can catalyze the synthesis of cmo5U from Cx-SAM in the presence of excess SAM, 10-μM Cx-SAM and 2-mM SAM were used for alkyltransfer assays and analyzed by LC-MS. cmo5U was detected after 20 min, whereas a trace amount of mo5U was detected only after 2 h. Therefore, the Cx-SAM-dependent carboxymethylation outcompetes the SAM-dependent methylation when both Cx-SAM and SAM are present at concentrations roughly an order of magnitude above their respective equilibrium dissociation constants (*K*_d_s; see the Discussion below). Using an LC-MS isotopic ratio-based method (17), we determined the *in vivo* concentration of Cx-SAM in *E. coli* K-12 to be 0.5 ± 0.1 μM. Thus, the Cx-SAM:SAM ratio used in this assay (1:200) roughly reflects the relative cellular abundance (i.e. 0.5 and 200 μM for Cx-SAM and SAM, respectively), supporting the biological relevance of the *in vitro* experiments.

### *In vivo* and *in vitro* activity of mutant CmoB

The three residues proposed to be responsible for recognition of the *S*-carboxymethyl moiety in Cx-SAM, Lys-91, Tyr-200 and Arg-315 were each mutated to alanine and *in vivo* activities were examined as described for wild-type CmoB. Somewhat unexpectedly, R315A and Y200A exhibited *in vivo* cmo5U synthetic activities similar to that of the wild type. However, K91A did not direct the synthesis of cmo5U after 24 h of induction, although mo5U was detected 3 h after induction of mutant expression. K91A was further analyzed *in vitro* by incubating mutant enzyme with SAM and total tRNA from Δ*cmoB* cells, which resulted in an MS signal corresponding to mo5U appearing between 1 and 2 h. No significant cmo5U signal was detected after 24 h when Cx-SAM was utilized as the alkyl donor, consistent with the *in vivo* results.

### ITC assay

The thermodynamics of wild-type and mutant CmoB interactions with Cx-SAM and SAM were examined by ITC (Table 1 and Supplementary Figure S8). The binding isotherms of both ligands are monophasic, indicating the presence of a single class of binding site for each ligand, except for the K91A mutant with Cx-SAM, where multi-phasic behavior was observed. The interaction between wild-type CmoB and Cx-SAM is characterized by an apparent equilibrium association constant (*K*) of 2.5 ± 0.2 × 10^6^ M^−1^ (*K*_d_ of 0.40 ± 0.02 μM), and is primarily enthalpically driven with Δ*H°* = −7.88 ± 0.05 kcal·mol^−1^, Δ*S°* = 1.92 ± 0.21 cal·mol^−1^·deg^−1^ (*T*Δ*S°* = 0.55 ± 0.06 kcal·mol^−1^) and Δ*G°* = −8.44 ± 0.03 kcal·mol^−1^. The interaction between the wild-type CmoB and SAM is much weaker with a *K* of 4.8 ± 2.0 × 10^3^ M^−1^ (*K_d_* of 0.21 ± 0.09 mM), Δ*H°* = −0.62 ± 0.12 kcal·mol^−1^, Δ*S°* = 14.7 ± 0.9 cal·mol^−1^·deg^−1^ (*T*Δ*S°* = 4.23 ± 0.27 kcal·mol^−1^) and Δ*G°* = −4.85 ± 0.24 kcal·mol^−1^. The thermodynamic parameters for the interactions with SAM could not be determined as confidently as those for Cx-SAM due to the low affinity, as evidenced by the relatively large uncertainties. While the ‘*c*-value’ of Cx-SAM (*c* = 125), defined as *N*·*K*·[CmoB]_cell_, where *N* and *K* represent the stoichiometry and the association constant, respectively, is in the optimal range for reliable determination of thermodynamic parameters by ITC, that of SAM (*c* = 2) is only marginally within the recommended range (1<*c*<1000) (26).

**Table 1.** Thermodynamics of the interaction of CmoB with Cx-SAM or SAM determined from ITC

	Wild type		K91A		Y200A		R315A	
	Cx-SAM	SAM	Cx-SAM	SAM	Cx-SAM	SAM	Cx-SAM	SAM
*K* (M^−1^)^a^	2.5 ± 0.2 x 10^6^	4.8 ± 2.0 x 10^3^	9.0 ± 1.8 x 10^3^	9.1 ± 0.2 x 10^4^	5.5 ± 0.8 x 10^5^	N.D.^e^ (< 8.4 × 10^3^)	4.7 ± 0.3 x 10^5^	7.1 ± 0.4 x 10^3^
*K*_d_ (μM)	0.40 ± 0.03	208.3 ± 86.8	110.9 ± 22.0	11.0 ± 0.2	1.8 ± 0.3	> 119	2.1 ± 0.2	140.2 ± 7.3
Δ*H*° (kcal/mol)^a^	−7.9 ± 0.1	−0.6 ± 0.1	−16.5 ± 1.6	−6.4 ± 0.1	−7.3 ± 0.1	N.D.	−8.5 ± 0.1	−11.1 ± 0.2
Δ*G*° (kcal/mol)^b^	−8.4 ± 0.1	−4.9 ± 0.2	−5.2 ± 0.1	−6.5 ± 0.1	−7.6 ± 0.1	>−5.1	−7.5 ± 0.1	−5.1 ± 0.1
Δ*S*°(cal/mol/deg)^c^	1.9 ± 0.2	14.7 ± 0.9	−39.3 ± 5.4	0.4 ± 0.1	0.8 ± 0.5	N.D.	−3.5 ± 0.3	−21.0 ± 0.8
Specificity ratio^d^	5.2 ± 2.2 x 10^2^		9.9 ± 2.0 x 10^−2^		> 65		6.6 ± 0.6 x 10	

^a^The binding constant (*K*) and enthalpy (Δ*H*°) were determined by fitting each titration curve to a single binding site (1:1) model.

^b^Δ*G*° = −*RT* lnK where *R* is the gas constant and *T* is the absolute temperature.

^c^Δ*S*° = (Δ*H*°−Δ*G*°)/*T*.

^d^Specificity ratio = *K*(Cx-SAM)/*K*(SAM).

^e^The binding constant of SAM was too weak to be determined by ITC (i.e. *c* = *K*[SAM] < 1 where [SAM] = 118.8 μM).

Cx-SAM binding affinities of the Y200A and R315A mutants were slightly reduced (4–5-fold) compared to the wild type, with *K*s (Cx-SAM) for Tyr-200 and Arg-315 of 5.5 ± 0.8 × 10^5^ and 4.7 ± 0.3 × 10^5^ M^−1^ (*K_d_*s of 1.8 ± 0.3 and 2.1 ± 0.2 μM), respectively. The relative binding specificity (defined as *K* (Cx-SAM)/*K* (SAM) in Table 1) for these mutants favors Cx-SAM by ∼65-fold, which is significantly less than the 520-fold discrimination exhibited by wild-type CmoB. This trend is reversed in K91A, which due to the combination of decreased affinity toward Cx-SAM (*K* = 9.0 ± 1.8 × 10^3^ M^−1^or *K_d_* = 111 ± 22μM) and increased affinity toward SAM (*K* = 9.1 ± 0.2 × 10^4^ M^−1^ or *K*_d_ = 11 ± 0.2 μM) results in relative binding specificity of ∼0.1. Notably, multiphasic binding pattern behavior was observed in the titration of K91A with Cx-SAM (Supplementary Figure S8), which suggests complex cooperative binding of ligands to the four sites in tetrameric K91A mutant protein. Detailed analysis of the ITC data of K91A and Cx-SAM is described in Supplementary information.

## DISCUSSION

### Substrate specificity of CmoB: SAM versus Cx-SAM

Our biochemical, genetic and structural data demonstrate that the primary *in vivo* function of CmoB is as a carboxymethyl transferase in the biosynthesis of cmo5U-modified tRNAs. The *in vitro* rate of SAM-dependent transmethylation catalyzed by CmoB appears to be negligible compared to that of the Cx-SAM-dependent carboxymethylation, with no significant SAM-dependent methylation activity observed in the presence of Cx-SAM. This behavior is due, in part, to the ∼500-fold difference in binding affinities between Cx-SAM and SAM for CmoB. However, the *K_d_*s for the Cx-SAM-CmoB (0.4 μM) and SAM-CmoB (200 μM) interactions are almost equivalent to the *in vivo* concentrations of Cx-SAM (0.5 μM) and SAM (180μM). Based solely on these affinities (measured in the absence of tRNA) and the relevant *in vivo* concentrations, the CmoB catalytic sites should be equally occupied by Cx-SAM and SAM; thus, additional contributions to selectivity must arise from chemical and/or physical steps occurring subsequent to substrate binding in order to achieve the product distribution observed *in vivo* (i.e. predominately cmoU-tRNA). Other methyltransferases typically exhibit significantly lower activities toward SAM-analogs compared to SAM, as the consequence of steric interference caused by the bulky side chain installed at the sulfonium center of the SAM analogs. CmoB is thus unique within the SDMT superfamily in that it preferentially utilizes a SAM analog and catalyzes an unprecedented alkyltransfer reaction.

The CmoB crystal structures offer insights into the determinants responsible for this selectivity. Key structural elements recognizing the *S*-carboxymethyl group of Cx-SAM are Lys-91, Tyr-200 and Arg-315 of CmoB, which are well positioned for binding anionic moieties such as sulfate or carboxylate functionalities as observed in the apo- and Cx-SAM bound structures, respectively. The side chain of Lys-91 exhibits conformational plasticity, interacting with the active site sulfate anion and a main chain carbonyl in the apo-structure. However, occupancy of the catalytic site with the bulkier Cx-SAM (or SAM) requires movement of the Lys-91 side chain in order to accommodate the amino acid portion of the ligand. This rearrangement may afford favorable electrostatic interactions with Cx-SAM given the close proximity of the ligand carboxylate; however, in the case of SAM, the positively charged sulfonium center may act as an anti-determinant to binding due to an unfavorable electrostatic interaction with the ϵ-amino group of Lys-91. Our *in vivo* and *in vitro* kinetic studies show that Lys-91 is indeed critical for Cx-SAM discrimination, as the K91A mutation resulted in the complete loss of the Cx-SAM-dependent carboxymethyltransfer activity. The K91A mutant displayed a SAM-dependent methyltransfer activity, resulting in mo5U formation, comparable (or somewhat greater) to that of the wild type, indicating that the lack of Cx-SAM-dependent carboxymethyltransfer activity is not caused by misfolding of the mutant, but is the consequence of reduced binding affinity and/or ineffective catalysis. This observation is supported by the equilibrium binding assays, where the elevated *K*_d_ for Cx-SAM (110 μM) greatly exceeds the physiological concentration of Cx-SAM (0.5 μM), while the affinity for SAM (*K*_d_ = 11 μM) is considerably more favorable and well below the cellular level of SAM (180 μM). The complete roles of Tyr-200 and Arg-315 in CmoB function and Cx-SAM/SAM discrimination remain to be resolved, as the ligand binding properties of the alanine mutants were only modestly affected (4–5-fold decreased). However the relative binding specificity could be reduced by as much as an order of magnitude for these mutants relative to wild type, which might represent a significant mechanistic contribution to the observed product distribution.

### Reaction mechanism of CmoB

SAM-dependent methyltransfer reactions occur via an S_N_2 mechanism, typically requiring nucleophile activation by deprotonation prior to in-line attack on the *S*-methyl group of SAM. CmoB residues in the vicinity of Cx-SAM that might act as a general base for activation of the 5-hydroxyl group of ho5U are His-201 and Glu-104, at distances of 7.5 and 5.2 Å from the electrophilic carbon center of *S*-carboxymethyl group of the ligand, respectively. Mutant enzymes in which each of these residues was changed to alanine were still active in the production of mo5U and cmo5U *in vivo*, suggesting that activation of the nucleophile is achieved through a CmoB-independent mechanism. The p*K*_a_ of the hydroxyl group on the pyrimidine ring of 5-hydroxy-2′-deoxyuridine is reported to be 7.68 in water (27), indicating that a significant fraction of the hydroxyl group of ho5U within tRNA will exist in a deprotonated and reactive form under physiological conditions. Thus, the intrinsic properties of ho5U might be sufficient to support the biologically relevant nucleophilic attack on Cx-SAM without further activation by CmoB functionalities. The possibility of substrate-assisted catalysis, involving activation of the incipient 5-hydroxy nucleophile via the carboxyl group of the *S*-carboxymethyl moiety of the Cx-SAM, cannot be ruled out. Participation of the Cx-SAM *S*-carboxymethyl functionality could also contribute to the preferential formation of cmo5U over mo5U, which is derived from SAM, although it is uncertain whether the stringent geometric requirements of the in-line S_N_2 alkyl-transfer mechanism would allow such a contribution.

The contributions of CH^…^O hydrogen bonding interactions to recognition and presentation of the *S*-methyl group have been reported for the S_N_2-type reactions catalyzed by lysine methyltransferases (22,28); however, no equivalent interactions are observed in CmoB. There are no residues positioned to form CH^…^O hydrogen bonds with the methylene of the S-carboxymethyl group; instead it appears that interactions involving the side chains of the three conserved residues (K91, Y200 and R315) with the Cx-SAM carboxyl group synergize to fine-tune the geometry of the *S*-carboxymethyl group for the biologically optimal nucleophilic attack. In addition, backbone carbonyl groups from Met-196 and Gly-197 engage the two methylene carbons flanking the sulfonium center through potential CH^…^O hydrogen bonds, which may further contribute to recognition and orientation of Cx-SAM.

### Biological significance

Our genetic and *in vitro* biochemical assays are consistent with a model in which the *in vivo* formation of mo5U arises from the SAM-dependent methyltransferase activity of CmoB. However, the significantly higher activity of CmoB in the biosynthesis of cmo5U, relative to that of mo5U, indicates that the predominant biological function of CmoB is the Cx-SAM-dependent carboxymethylation of wobble ho5U in tRNA, with SAM-dependent methyltransfer likely representing a side reaction. Sequence analysis shows widespread conservation of CmoB orthologs among the Gram-negative proteobacteria, as well as in the some *Verrucomicrobia, Acidobacteria and Cyanobacteria* (Supplementary Figure S9A). A complete description of the *in vivo* functions of the cmo5U modification remains to be fully defined, as no obvious growth defects have been reported for either the *cmoA*- or *cmoB*-knockout mutants in *E. coli K-12* (29). However, *cmoA* was classified as one of the candidate fitness genes in uropathogenic *E. coli CFT073*, which were required for optimal survival in the mouse spleen (30). In addition, fairly strong fitness defects have been reported for *cmoA* and *cmoB* mutants in *Shewanella oneidensis MR-*1 when grown at pH 6 or using N-acetylglucosamine as a carbon source (31).

It was shown that Cx-SAM remained tightly bound in the active site of recombinant CmoA after multiple chromatography steps and crystallization (14). Given this high affinity, the relatively low *in vitro* activity of CmoA is likely due to significant product inhibition, which may reflect regulatory mechanisms controlling the biosynthesis of Cx-SAM destined for wobble uridine modification. Regulation of the cellular pool of Cx-SAM might minimize unwanted carboxymethylation of unintended protein or small molecule targets via other methyltransferases. Such off-target effects could result in decreased fitness, although other potential biological acceptors of the carboxymethyl moiety remain to be discovered. It is interesting that the *K*_d_ for the Cx-SAM-CmoB interaction is close to the cellular concentration of Cx-SAM at stationary phase, as might be expected for efficient catalysis. *cmoA* and *cmoB* are co-conserved, residing immediately adjacent to each other in resident genomes, and are predicted to form a two-cistron operon in most γ-proteobacteria (Supplementary Figure S9B) (13). Therefore, tight regulation of cmo5U modification might be achieved at both biochemical and genetic levels.

Cx-SAM is the first example of a naturally occurring SAM derivative with a biological function. Functional diversification of the *S*-methyl group of SAM has been actively pursued to support *in vitro* chemical manipulation after transfer to the biological acceptor of interest. SAM analogs with an extended *S*-alkyl group containing a double or triple bond have been exploited in DNA alkylation reactions by DNA methyl transferases (32,33), labeling of a histone H3 peptide with a protein methyl transferase (34), modification of tRNA^phe^ by Trm1 (35) and alkylation of pre-mRNA by C/D box ribonucleotide methyltransferase (36). The introduction of an alkynyl functionality on the modified macromolecules allowed for further labeling through click chemistry with an azide-derivatized tag including an azide-biotin (34), azide-fluorescent probe (35) or azide-FLAG epitope (37). However, because these methyltransferases have evolved to bind SAM, bulky SAM-analogs frequently display lower and non-specific reactivities. No *in vivo* activities have been reported for these bioorthogonal species, presumably due to a limited cell permeability, unfavorable chemical stability and low activity.

CmoA and CmoB are the first documented synthase and transferase for Cx-SAM, and represent unique cases of functional diversification within the SAM-dependent methyltransferase superfamily. This description of Cx-SAM biosynthesis and utilization expands the metabolic and biological contributions of SAM-based biochemistry, and suggests the possible existence of other biologically relevant SAM analogs and their cognate synthases and alkyltransferases.

## ACCESSION NUMBERS

Atomic coordinates and structure factors for the apo and liganded crystal structures of CmoB are deposited in the Protein Data Bank under the accession codes 4QNX and 4QNV (P6122) and 4QNU (P21212), respectively.

## SUPPLEMENTARY DATA

Supplementary Data are available at NAR Online.

SUPPLEMENTARY DATA
