# Supplementary material for: Determinants of the CmoB carboxymethyl transferase utilized for selective tRNA wobble modification
Source: Nucleic Acids Res. 2015 Apr 8;43(9):4602–13. doi: 10.1093/nar/gkv206 (PMC4482062; doi:10.1093/nar/gkv206)
Supplement: SUPPLEMENTARY DATA [file supp_43_9_4602__index.html]

Determinants of the CmoB carboxymethyl transferase utilized for selective tRNA wobble modification — Determinants of the CmoB carboxymethyl transferase utilized for selective tRNA wobble modification — SUPPLEMENTARY DATA 

# Determinants of the CmoB carboxymethyl transferase utilized for selective tRNA wobble modification

## SUPPLEMENTARY DATA

**Files in this Data Supplement:**

- SUPPLEMENTARY DATA
