# Supplementary material for: Determinants of the CmoB carboxymethyl transferase utilized for selective tRNA wobble modification
Source: Nucleic Acids Res. 2015 Apr 8;43(9):4602–13. doi: 10.1093/nar/gkv206 (PMC4482062; doi:10.1093/nar/gkv206)
Supplement: SUPPLEMENTARY DATA [file supp_gkv206_nar-02710-r-2014-File007.docx]

**Supplementay Information**

**Determinants of the CmoB carboxymethyl transferase utilized for selective tRNA wobble modification**

Jungwook Kim^1*^, Hui Xiao^2^, Junseock Koh^3^, Yikai Wang^4^, Jeffrey B. Bonanno^1^, Keisha Thomas^1^, Patricia C. Babbitt^5^, Shoshana Brown^5^, Young-Sam Lee^6^ and Steven C. Almo^1,7*^

**Interpretation of the macroscopic binding constants for the interaction between K91A mutant and Cx-SAM**

The biphasic feature observed in the titration of K91A with Cx-SAM suggests cooperative (positive or negative) binding of ligands to the four sites in tetrameric K91A CmoB. The titration curve was analyzed using macroscopic binding constants and enthalpies describing binding of *n*-molecules of Cx-SAM to tetrameric mutant CmoB. Initially, the titration curve was analyzed with all possible values of *n* (=1~4). However, K_3_ converged to negative values with significantly large uncertainties, indicating that the population of tetrameric K91A with three ligands bound is negligible over the course of titration. Therefore, the titration curve was analyzed using three sets of binding constants and enthalpies (**Supplementary Table 2**). The fitted values of the macroscopic binding constants were compared to those expected for a case of non-cooperative binding. For both cooperative and non-cooperative binding, K_1_ is given by 4K_site_ where 4 accounts for the number of ligand binding sites in teterameric CmoB (i.e., statistical factor) and K_site_ describes the “intrinsic” affinity of each site. Then, K_site_=3.6x10^4^ M^-1^. The fitted value of K_2_ is ~ 8.4-fold higher than that calculated for the case of non-cooperative binding (6K_site_^2^=7.7x10^9^ M^-2^), indicating positive cooperativity involved in binding of the second Cx-SAM. Since the population of K91A with three Cx-SAMs bound is negligible, binding of the third Cx-SAM is deduced to be negatively cooperative. However, the fitted value of K_4_ is ~ 98.5-fold higher than that calculated for the case of non-cooperative binding (K_site_^4^=1.7x10^18^ M^-4^), indicating that binding of the fourth Cx-SAM involves highly positive cooperativity (The third and the fourth Cx-SAMs simultaneously bind tetrameric K91A mutant CmoB).

A possible molecular explanation for such heterogeneous cooperativity involved in the interaction between K91A and Cx-SAM is the presence of (at least) two different types of interface between the subunits in tetrameric K91A which is consistent with the structural model of the CmoB tetramer. In this model (**Supplementary Figure 9**), the four ligand binding sites are initially equivalent, but binding of the first ligand to any of these four sites differentially affects (increases and decreases) the affinities of the neighboring sites for the second ligand through the different subunit interfaces. This model is a variation of the classical “square” model used in the analysis of the interaction between hemoglobin and oxygen(2) and provides a molecular description of the fitted macroscopic binding constants in terms of the intrinsic affinity (K_site_) and two types of cooperativity (ω_+_ and ω_-_): K_1_=4K_site_; K_2_=2K_site_^2^(1+ω_+_+ω_-_); K_4_=K_site_^4^(ω_+_ω_-_)^2^. Then, K_site_=3.6x10^4^ M^-1^, ω_+_=23.8, and ω_-_=0.4. The value of ω is equivalent to a fold-change in the Cx-SAM affinity of a binding site in tetrameric K91A when its neighboring site is already occupied by Cx-SAM.

|  | Sel-Met | Apo | Cx-SAM1 | Cx-SAM2 |
| --- | --- | --- | --- | --- |
| **Data collection** |  |  |  |  |
| Wavelength (Å) | 0.9792 | 1.0745 | 1.0745 | 1.0745 |
| Space group | *P2_1_* | *C2* | *P2_1_2_1_2* | *P6_1_22* |
| Cell dimensions |  |  |  |  |
| *a*, *b*, *c* (Å) | 61.79,328.78,184.90 | 75.57,185.80,75.01 | 184.80,347.55,59.42 | 82.13,82.13,432.52 |
| *α*, *β*, *γ* (°) | 90,93.85,90 | 90,101.54,90 | 90,90,90 | 90,90,120 |
| Resolution (Å) | 50-3.11 (3.16-3.11) | 50-2.63 (2.68-2.63) | 50-2.58 (2.62-2.58) | 50-2.64 (2.69-2.64) |
| *R*_merge_ | 0.080 (0.498) | 0.078 (0.84) | 0.13 (0.74) | 0.144 (0.99) |
| *I*/σ*I* | 14.3 (2.7) | 18.1 (2.0) | 8.8 (2.0) | 15.5 (4.0) |
| Completeness (%) | 89.4 (83.7) | 98.9 (99.7) | 85.1 (87.1) | 99.2 (100) |
| Redundancy | 3.5 (3.6) | 5.1 (5.0) | 2.8 (3.1) | 8.5 (8.8) |
|  |  |  |  |  |
| **Refinement** |  |  |  |  |
| Resolution (Å) |  | 47.54-2.62 | 43.91-2.60 | 43.08-2.64 |
| No. reflections |  | 30,170 | 95,523 | 25,243 |
| *R*_work_/ *R*_free_ |  | 0.20/0.25 | 0.21/0.26 | 0.22/0.27 |
| No. atoms |  |  |  |  |
| Protein |  | 5,170 | 20,633 | 5,185 |
| Ligand/ion |  | 80 | 290 | 70 |
| Water |  | 88 | 339 | 40 |
| B-factors |  |  |  |  |
| Average |  | 47.10 | 46.90 | 49.00 |
| Protein |  | 46.90 | 47.10 | 49.00 |
| Water |  | 42.60 | 31.10 | 36.50 |
| R.m.s deviations |  |  |  |  |
| Bond lengths (Å) |  | 0.009 | 0.009 | 0.011 |
| Bond angles (º) |  | 1.188 | 1.286 | 1.536 |
| Ramanchandran Analysis |  |  |  |  |
| Favored (%) |  | 98 | 95 | 95 |
| Allowed (%) |  | 2 | 5 | 5 |
| Outliers (%) |  | 0 | 0 | 0 |

**Supplementary Table I.** Crystallographic statistics.

**Supplementary Table 2.** Analysis of the interaction between CxSAM and the K91A mutant using macroscopic binding constants and enthalpies^a^

| K_1_ (M^-1^) | ∆H_1_° (kcal/mol) | K_2_ (M^-2^) | ∆H_2_° (kcal/mol) | K_4_ (M^-4^) | ∆H_4_° (kcal/mol) |
| --- | --- | --- | --- | --- | --- |
|  |  |  |  |  |  |
| 1.4 (± 0.3) x 10^5^ | - 17.4 (± 1.0) | 6.5 (± 1.4) x 10^10^ | - 26.4 (± 0.3) | 1.6 (± 0.5) x 10^20^ | - 37.2 (± 0.2) |

^a^In this model, the interaction is described by a set of binding events where *n-*molecules of Cx-SAM bind tetrameric CmoB (K91A) with the corresponding binding constants (K*_n_*) and enthalpies (∆H*_n_*°). Initially, the titration curve was analyzed by this model including all possible values of *n* (=1~4). However, K_3_ converged to negative values with significantly large uncertainties, indicating that the population of tetrameric CmoB (K91A) with three ligands bound is negligible over the course of titration. Therefore, the titration curve was analyzed using three sets of binding constants and enthalpies.


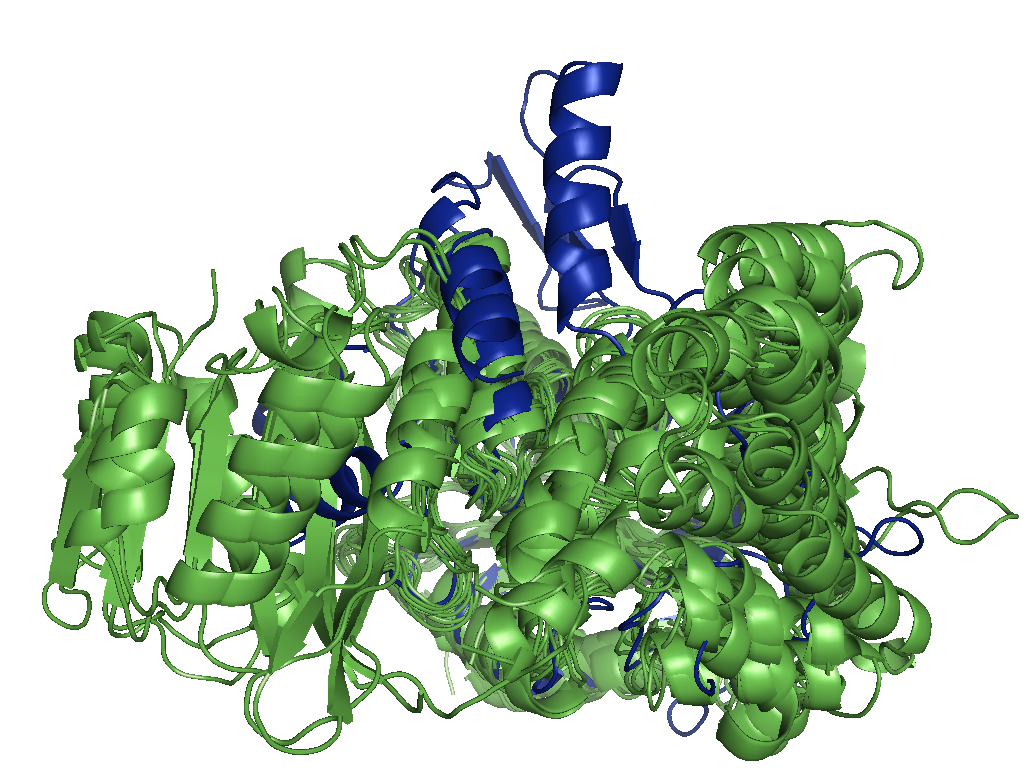


**Supplementary Fig 1. Structural homologues of CmoB.** Superposition of structures of CmoB and top 10 hits (green) in the structural homologue search in DALI database: PDB codes 2O57, 4F84, 3KKZ, 3BUS, 1XXL, 3T7S, 4FGZ, 3UJ7, 4INE, and 4KRI. Coordinates of Cx-SAM bound CmoB was used for search query, which is represented in blue. Note the segment composed of the N-terminal 100 residues of CmoB is not superimposable with the structures of other SDMT.

**Supplementary Fig 2**. **Conservation of amino acids residues among 818 bacterial CmoB.** Conservation of each residue is mapped onto the CmoB structure; highly conserved residues are displayed in red, followed by orange, yellow, green, light blue, and dark blue, which represents the most divergent residues. The map was generated by ConSurf Server (http://consurf.tau.ac.il/).


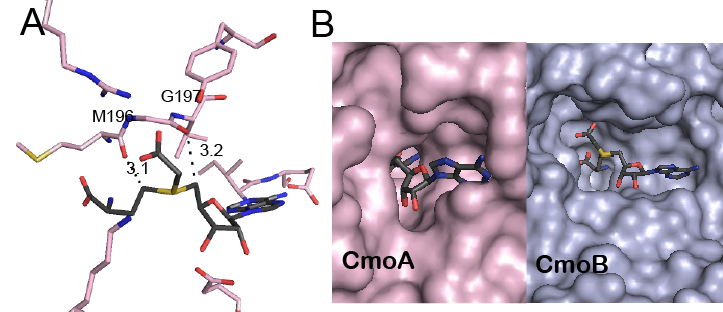


**Supplementary Fig 3. Cx-SAM bind site of CmoB.** A) Backbone carbonyl oxygen atoms of Met-196 and Gly-197 form CH**^…^**O hydrogen bond with Cx-SAM. B) Comparison of Cx-SAM binding pocket between CmoA and CmoB. Note that the S-carboxymethyl group is largely buried in the CmoA, whereas it is exposed to the solvent in CmoB.


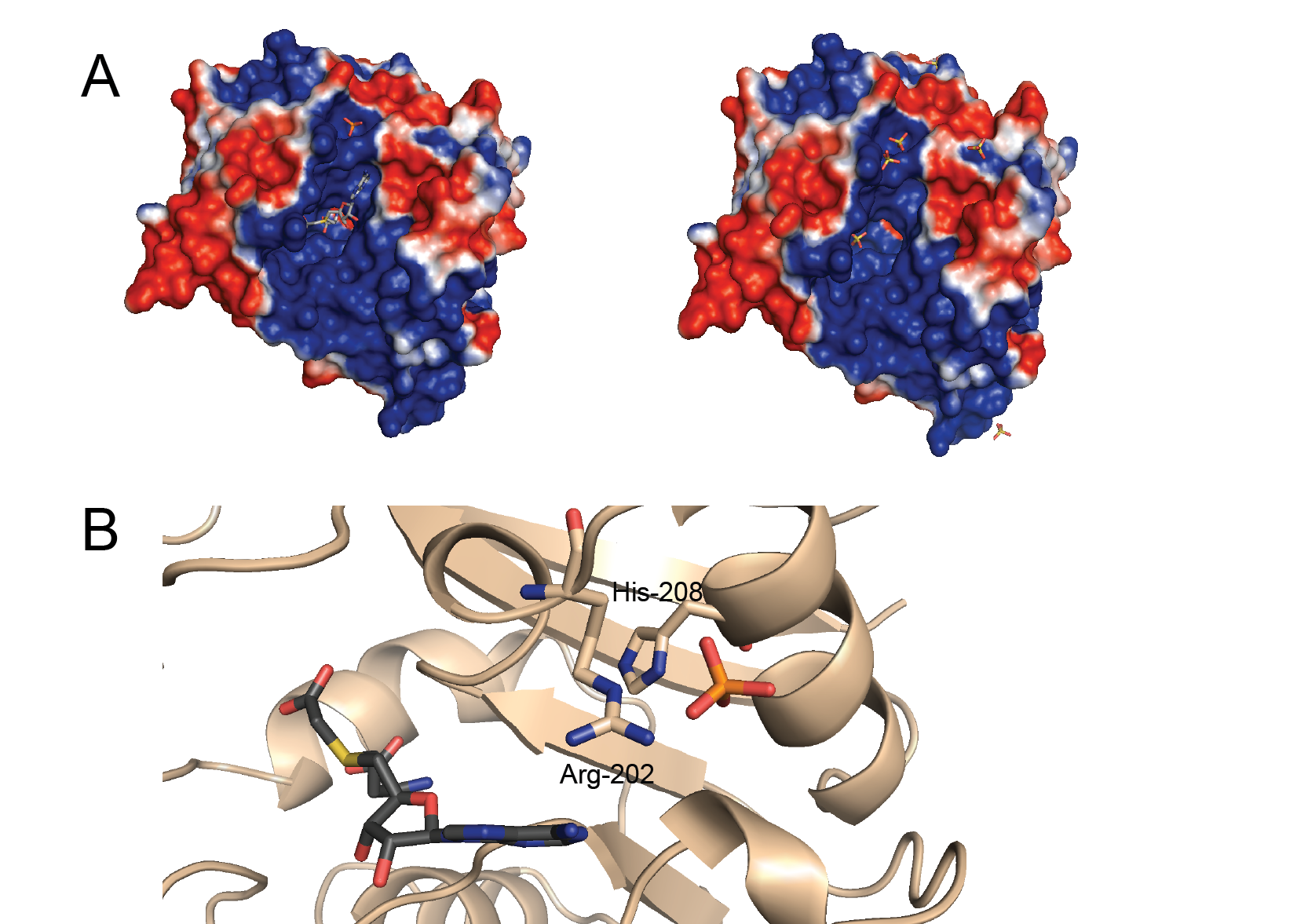


**Supplementary Fig 4. Electrostatic potential of the protein surface mapped on the CmoB structure.** Electrostatic surface potential displayed at contour levels of + kT/e (blue), or – kT/e (red), respectively. A) A monomer of the Cx-SAM bound structure where Cx-SAM and a phosphate ion are represented as sticks (left), and a monomer of the apo-structure with sulfate ions represented in sticks (right). B) The conserved phosphate identified in all subunits, forming polar interactions with Arg-202 and His-208. In the apo-structure, an equivalent sulfate ion is present instead in nearly identical pose.


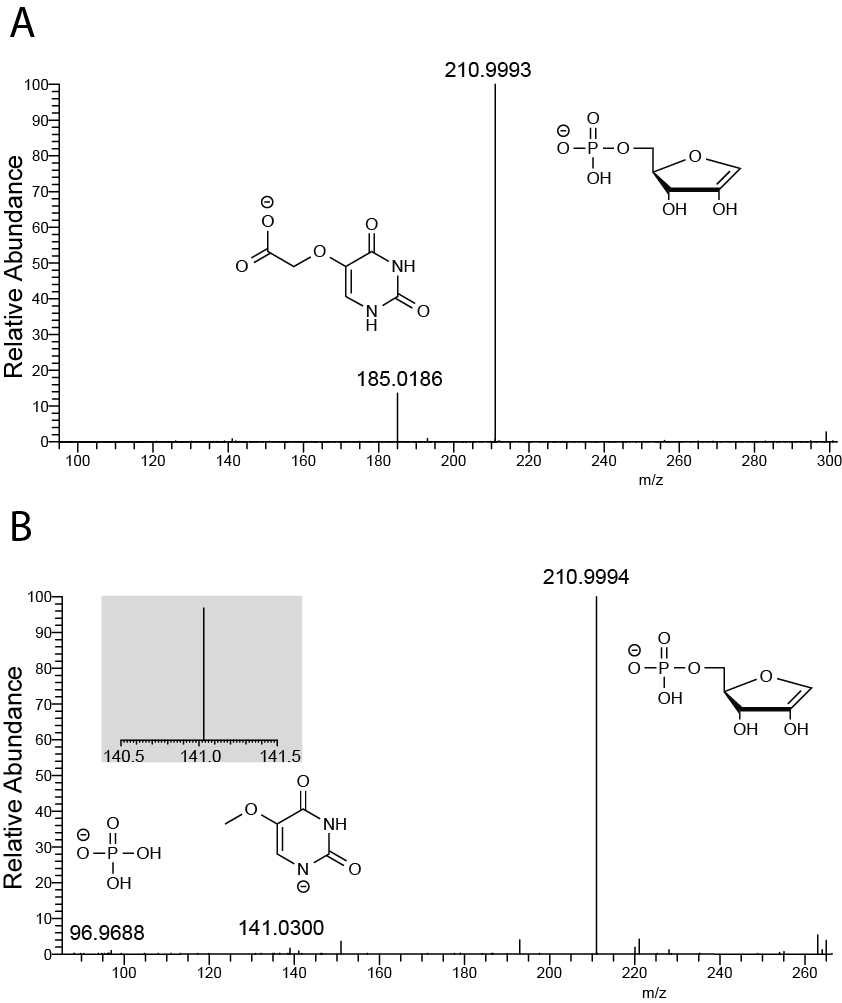


**Supplementary Fig 5. LC-MS/MS analyses of the total tRNA from wild type *E. coli*.** Wobble modification was analyzed after hydrolyzing *E. coli* total RNA (Sigma-Aldrich) with P1 nuclease followed by LC/MS/MS analysis in negative mode. A parent mass corresponding to A) cmo5UMP (calculated m/z = 397.0284), or B) mo5UMP (calculated m/z = 353.0386) was detected and further validated through subsequent fragmentation by collision induced dissociation. Inset in B) is a close-up of the MS/MS peak corresponding to 141.0300. Notably, mo5UMP was exclusively detected in *cmoA*- and *aroC*-mutants, chromatograms of which are similar to B). For comparison, MS/MS fragmentation of enzymatically generated mo5UMP is shown in Supplementary Fig 5-1. Plausible chemical structures of the fragments are shown next to the corresponding m/z peaks: calculated *m/z* of ribosyl phosphate (C_5_H_8_O_7_P^-^) = 211.0008, 5-oxyacetyluracil (C_6_H_5_N_2_O_5_^-^) = 185.0198, 5-methoxyuracil (C_5_H_5_N_2_O_3_^-^) = 141.0300, and phosphate (PO_4_H_2_^-^) = 96.9691.


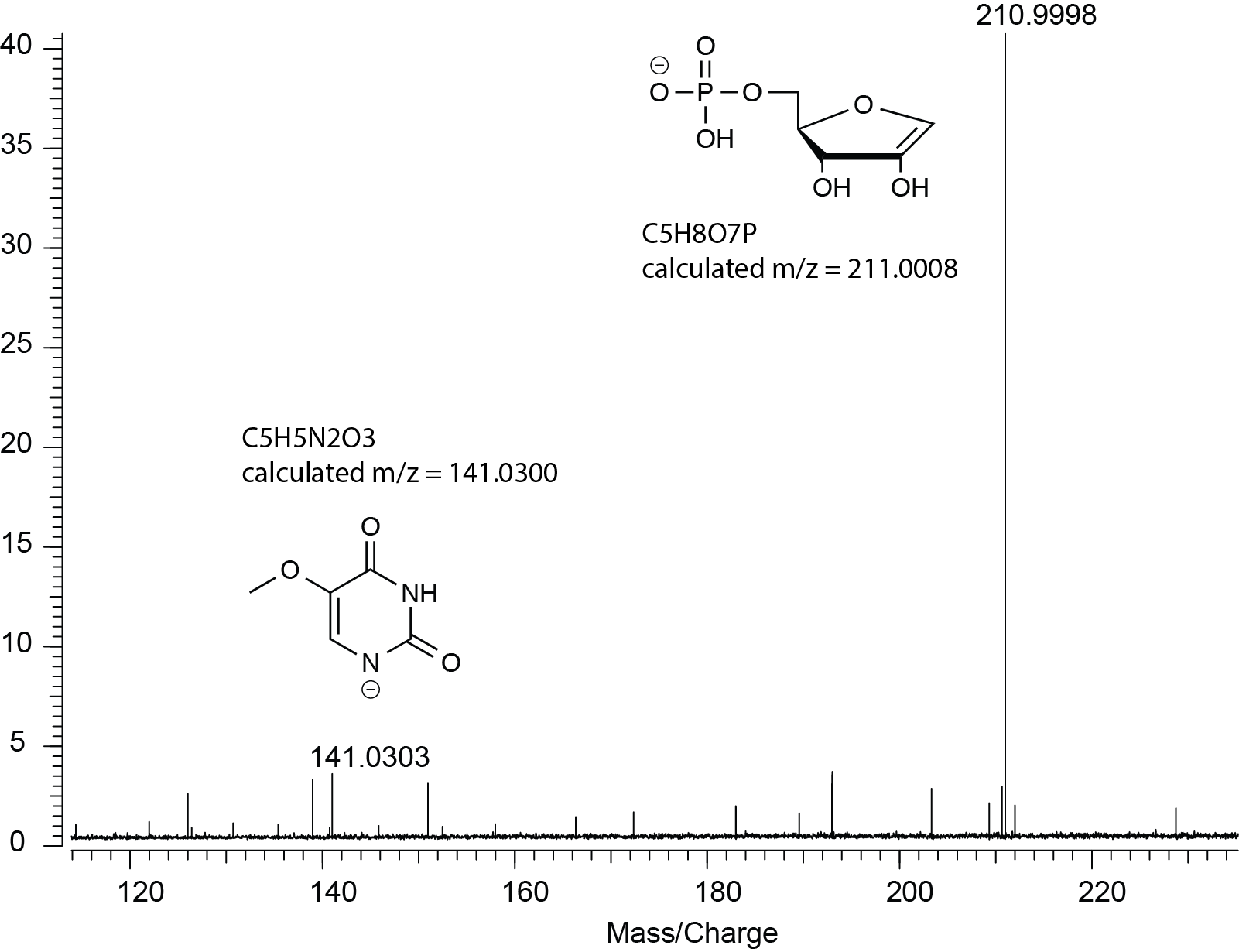


**Supplementary Fig 5-1. MS/MS analyses of an enzymatically synthesized mo5UMP standard.** A chemical standard for mo5UMP was enzymatically synthesized using uracil phosphoribosyltransferase (UPRT). A solution containing 20mM Tris (PH= 7.0), 4mM 5-methoxyuracil (Toronto Research Chemicals Inc.), 4mM 5-phospho-D-ribose 1-diphosphate (Sigma-Aldrich), 10mM MgSO_4_, and 0.1 mg/mL UPRT of *Vibrio cholerae*, was incubated overnight at room temperature. Formation of mo5UMP was confirmed by mass spectrometry, and the compound was purified from the reaction mixture by HPLC. MS/MS analyses were performed using 12T Agilent IonSpec FT-ICR-MS in negative ion mode.


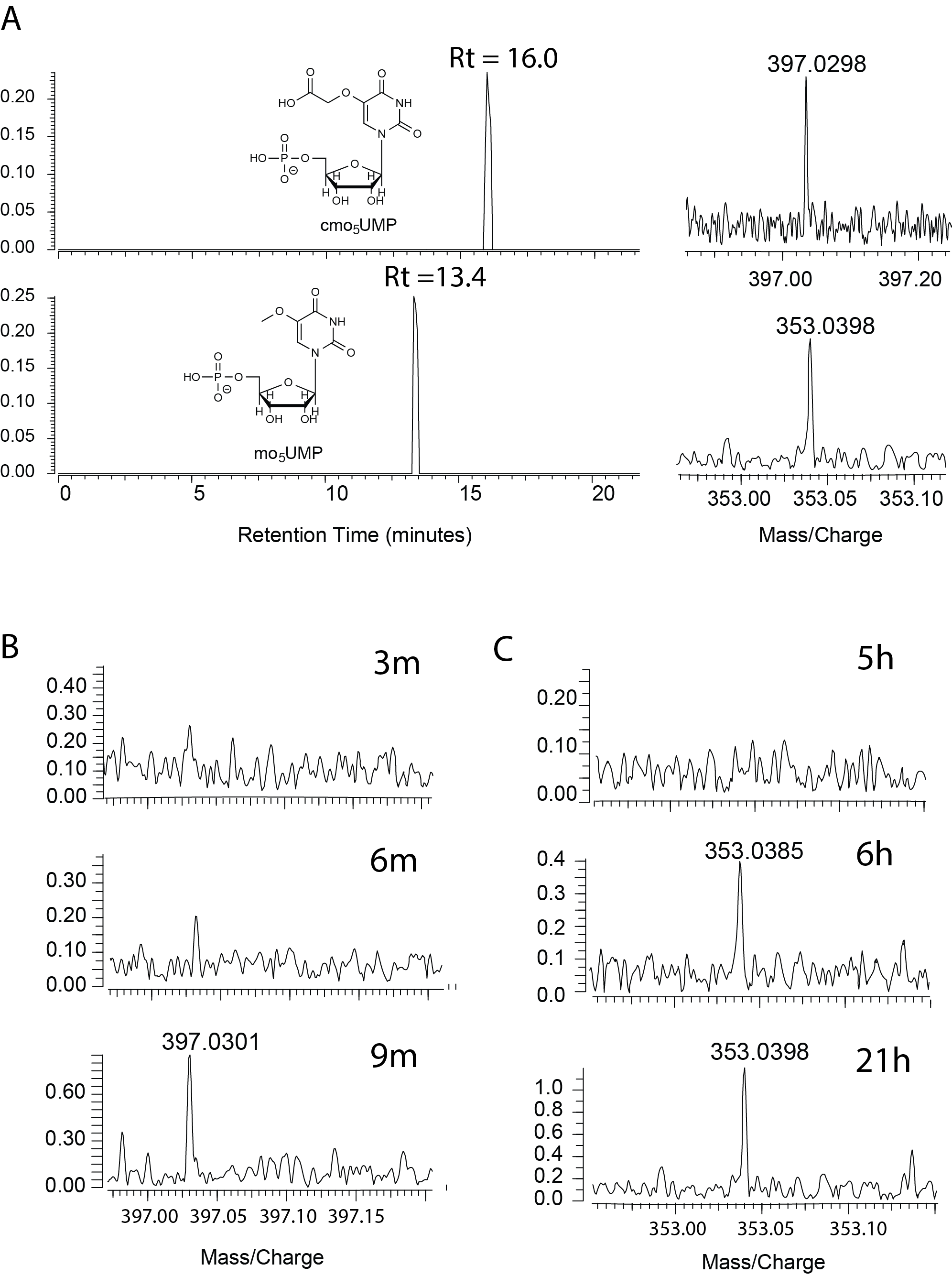


**Supplementary Fig 6. *In vitro* activity of the wild type CmoB.** The assay was quenched periodically by P1 nuclease digestion at 60°C for 1h prior to LC-MS analyses in negative ion mode. A) Representative HPLC elution and associated MS peaks of cmo5UMP (top) and mo5UMP (bottom), where Rt denotes the retention time. The traces of *m/z* corresponding to 397.03±0.01 (cmo5UMP), or 353.04±0.01 (mo5UMP) from *in vitro* reactions are displayed. Time dependent *in vitro* formation of cmo5UMP (B), or mo5UMP (C) by wild type CmoB was monitored by LC-MS. Note that an MS peak corresponding to cmo5UMP (calculated m/z = 397.0284) began to emerge after 9min, but not before 6min, validating the effectiveness of the quenching method.


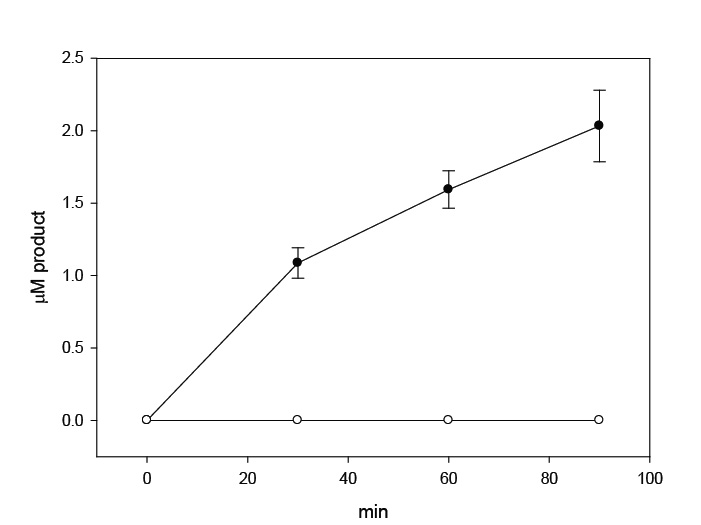


**Supplementary Fig 7. Time-course of the *in vitro* formation of alkylated tRNA by CmoB.** The wild type CmoB was incubated with [^14^methyl] SAM, ho5U containing tRNA, and with (white circles) or without (closed circles) Cx-SAM in methylation assay. An aliquot was withdrawn every 30min and radioactivity on tRNA was measured. Each value is an average of three independent measurements and error bars correspond to the standard deviation.


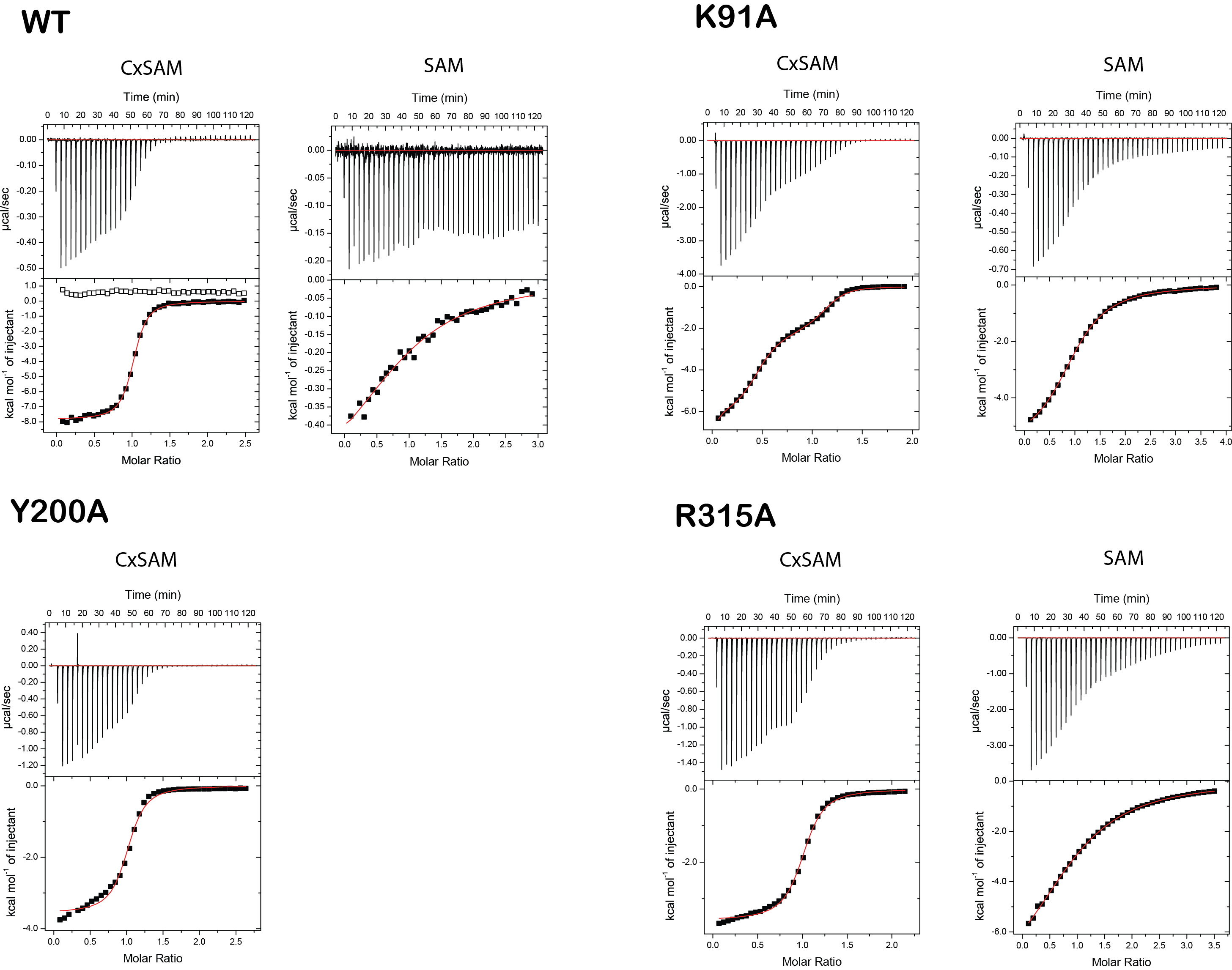


**Supplementary Fig 8. Interaction of CmoB with Cx-SAM and SAM monitored by isothermal titration calorimetry (ITC).** Raw heat signals generated by the titration of CmoB with multiple injections of a concentrated sample containing Cx-SAM or SAM are shown in the top panel as time traces. The raw heat signals were integrated over time, normalized per mol of ligand injected, and corrected for the heat of dilution to obtain heats of binding which are plotted in the bottom panel as a function of the molar ratio. The continuous lines are simulated titration curves from fitting the titration data to a single-site model except for K91A and Cx-SAM. The binding constant for Y200A with SAM was too weak to be determined.


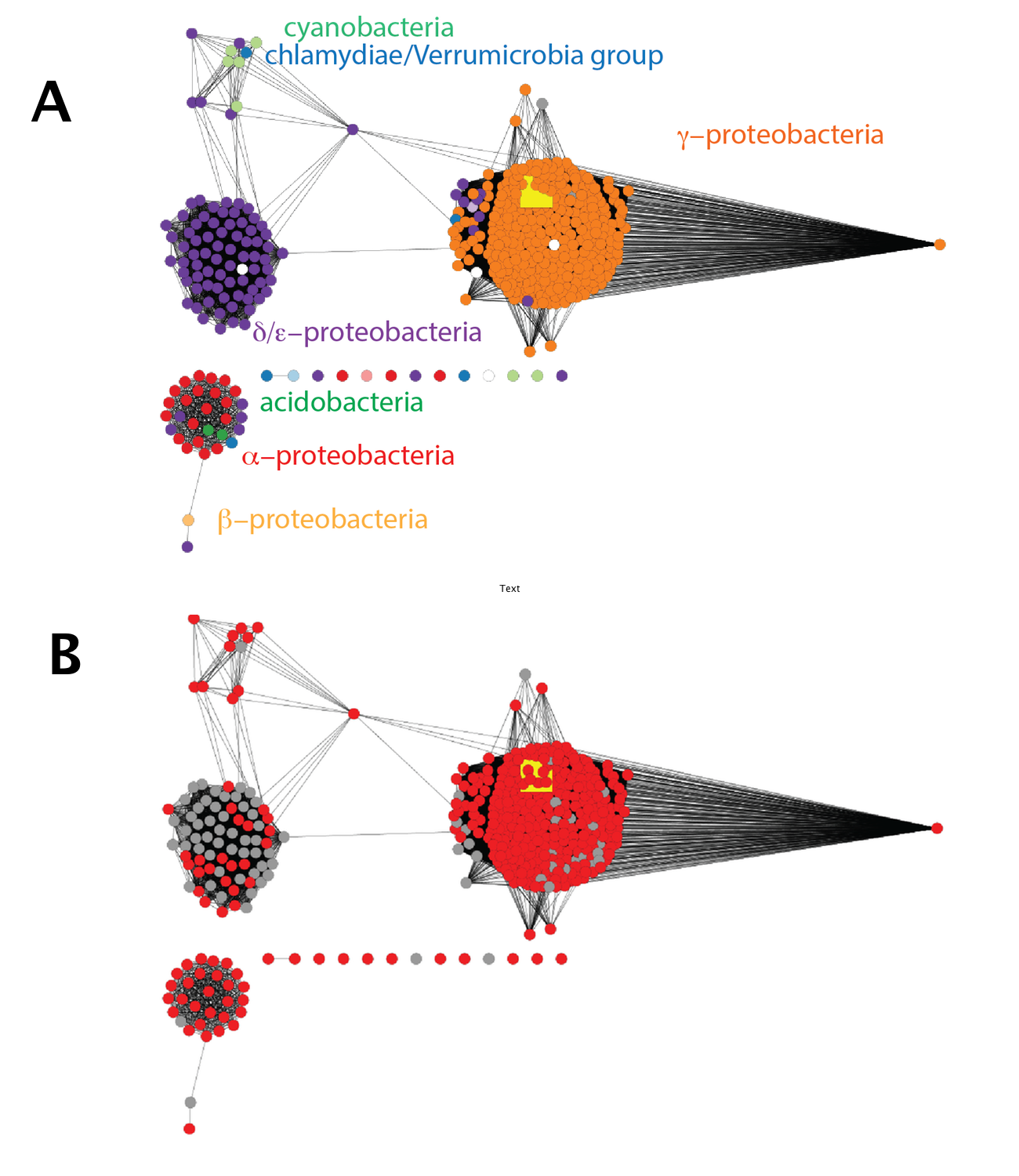


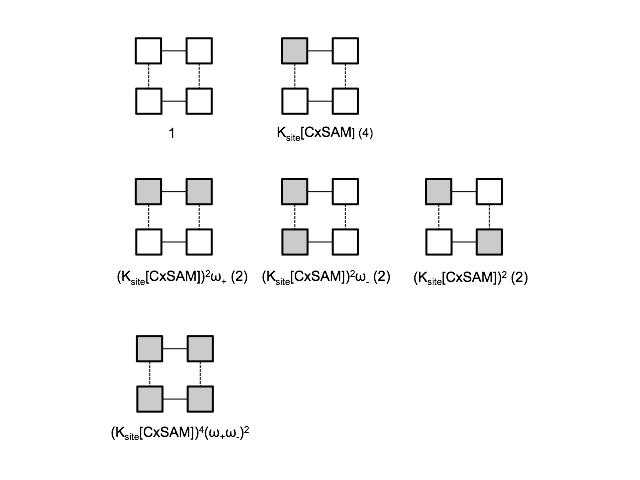


**Supplementary Fig 9.** **Cytoscape representation CmoB orthologs in the Pfam methyltransferase 9 family at an e-value cutoff of 1e-70.** Each node in the network represents a single sequence in the Pfam Methyltransf_9 family (obtained from the Structure Function Linkage Database (1)), and each edge represents the pairwise connection with the most significant BLAST E-value (better than the cut-off) connecting the two sequences. Connections between nodes are only shown if the e-value of the best Blast hit between two sequences is at least as good as the specified e-value cutoff. A yellow square denotes *E. coli* CmoB. A) Species distribution of *E. coli* CmoB relatives from the Pfam methyltrans_9 family. B) Red circles represent species co-appearance of CmoB and CmoA (in methyltrans_18).

**Supplementary Fig 10. Schematic illustration of a cooperative binding model for CmoB (K91A):Cx-SAM interaction.** Each open or filled square represents a CmoB (K91A) monomer with Cx-SAM free or bound, respectively. Solid and dashed lines represent positive and negative cooperativity between two adjacent subunits, respectively. All possible Cx-SAM bound states of tetrameric CmoB (K91A) are shown together with the corresponding statistical factor (reference state=free CmoB tetramer). The number in the bracket is the number of possible configuration for each Cx-SAM bound state.

**Supplementary Methods**

**Crystallization and experimental phasing of Se-Met apo-CmoB.**  Diffraction quality crystals were obtained by vapor diffusion by mixing 1μL of Se-Met labeled CmoB (10mg/mL in 20mM Tris-HCl (pH 8.5) and 150mM KCl) with 1μL of precipitant (1.1 M Ammonium tartrate dibasic (pH 7.0)) followed by equilibration against 70μL of the precipitant. Crystals were cryoprotected by addition of 25% glycerol, mounted in a nylon loop, and flash-cooled in liquid nitrogen. Single wavelength anomalous diffraction (SAD) data, consistent with space group P2_1_, were collected at 100K using an X-ray wavelength in the vicinity of the selenium absorption edge (λ=0.97920) on an ADSC QUANTUM 315 CCD detector at the NSLS beam line X29A and processed to 3.11Å resolution using HKL2000(3) (see **Supplementary Table 1**). An initial model of the selenium heavy atom substructure (70 out of 84 ordered sites) was determined using the SHELX suite of programs as implemented in the HKL2MAP interface(4). SAD phases were calculated using the automated protocol in PHASER(5), including heavy atom substructure completion via anomalous difference Fourier synthesis; this was followed by density modification using DM, which yielded phases of sufficient quality to yield helical features in the resulting electron density. Using a combination of the selenium sites and manually placed secondary structural elements, the non-crystallographic symmetry (NCS) operators interrelating the 12 monomers (arranged in 3 tetramers) were determined and improved using the programs MAMA and IMP(6). Additional density modification incorporating 12-fold NCS averaging using DM(7), yielded interpretable electron density for most of the CmoB molecule. This map allowed for an initial model to be built by PHENIX(8) and Coot(9).

**Crystallization and Structure Determination of apo-CmoB***.* Purified CmoB (0.3mM) was crystallized by sitting drop vapor diffusion at 21°C by mixing 1 μL of the protein with 1 μL of reservoir solution (1.26M ammonium sulfate, and 100mM HEPES/NaOH pH 7.5) and equilibrating over 0.1mL of reservoir solution. X-ray data were collected at 100K and a wavelength *λ* = 1.075Å on an ADSC QUANTUM 315 CCD detector at the NSLS beam line X29A and processed with HKL3000(10). Diffraction from apo-CmoB crystals was consistent with space group, C2 (**Supplementary Table I**), with two molecules per asymmetric unit. Molecular replacement was performed with MOLREP(11) using a model derived from the above Se-Met apo-CmoB data as the search model. Subsequent model building and refinement were performed with Coot and PHENIX.

**Crystallization and Structural Determination of Cx-SAM-bound CmoB***.*  0.5mM synthetic Cx-SAM was mixed with 0.3mM CmoB for co-crystallization. Crystals in two different space groups have been identified to contain Cx-SAM. Initial crystals of the CmoB:Cx-SAM complex were formed by mixing with crystallization solution containing 100mM HEPES/NaOH pH 7.5, 200mM MgCl2, and 30%(v/v) PEG400. These crystals exhibited diffraction consistent with space group P2_1_2_1_2, with eight monomers in the asymmetric unit. During the optimization of the condition above, a crystal form was identified in the identical reservoir solution with addition of 10mM TCEP:HCl. These crystals exhibited diffraction consistent with space group P6_1_22, with two monomers in the asymmetric unit. X-ray data were collected and processed as described above. Molecular replacement was performed using an apo-CmoB structure as the initial model with MOLREP. Subsequent model building and refinement were performed with Coot and REFMAC5(12).

**Supplementary References**

1. Akiva E*, et al.* (2014) The Structure-Function Linkage Database. *Nucleic Acids Res* 42(Database issue):D521-530.

2. Koshland DE, Jr., Nemethy G, & Filmer D (1966) Comparison of experimental binding data and theoretical models in proteins containing subunits. *Biochemistry* 5(1):365-385.

3. Otwinowski Z & Minor W (1997) Processing of X-ray diffraction data collected in oscillation mode. *Method Enzymol* 276:307-326.

4. Pape T & Schneider TR (2004) HKL2MAP: a graphical user interface for macromolecular phasing with SHELX programs. *J Appl Crystallogr* 37:843-844.

5. Mccoy AJ*, et al.* (2007) Phaser crystallographic software. *J Appl Crystallogr* 40:658-674.

6. Kleywegt RJRGJ (2001) Density modification:theory and practice. *Methods in Macromolecular Crystallography*, ed Johnson DTL (IOS Press, Amsterdam), pp 123-135.

7. Cowtan K (1994) *Joint CCP4 and ESF-EACBM Newsletter on Protein Crystallography* 31:34-38.

8. Afonine PV*, et al.* (2012) Towards automated crystallographic structure refinement with phenix.refine. *Acta Crystallogr D* 68:352-367.

9. Emsley P, Lohkamp B, Scott WG, & Cowtan K (2010) Features and development of Coot. *Acta Crystallogr D* 66:486-501.

10. Minor W, Cymborowski M, Otwinowski Z, & Chruszcz M (2006) HKL-3000: the integration of data reduction and structure solution - from diffraction images to an initial model in minutes. *Acta Crystallogr D* 62:859-866.

11. Vagin A & Teplyakov A (1997) MOLREP: an automated program for molecular replacement. *J Appl Crystallogr* 30:1022-1025.

12. Murshudov GN*, et al.* (2011) REFMAC5 for the refinement of macromolecular crystal structures. *Acta Crystallogr D* 67:355-367.
